# Renal cell carcinoma risk associated with lower intake of micronutrients

**DOI:** 10.1002/cam4.1639

**Published:** 2018-07-02

**Authors:** Cathryn H. Bock, Julie J. Ruterbusch, Andreana N. Holowatyj, Susan E. Steck, Alison L. Van Dyke, Won Jin Ho, Michele L. Cote, Jonathan N. Hofmann, Faith Davis, Barry I. Graubard, Kendra L. Schwartz, Mark P. Purdue

**Affiliations:** ^1^ Karmanos Cancer Institute Wayne State University School of Medicine Detroit MI USA; ^2^ Department of Population Health Sciences Huntsman Cancer Institute University of Utah Salt Lake City UT USA; ^3^ Epidemiology and Biostatistics The Cancer Prevention and Control Program Arnold School of Public Health University of South Carolina Columbia SC USA; ^4^ Division of Cancer Epidemiology and Genetics National Cancer Institute Bethesda MD USA; ^5^ Department of Oncology Sidney Kimmel Comprehensive Cancer Center Johns Hopkins School of Medicine Baltimore MD USA; ^6^ School of Public Health University of Alberta Edmonton AB Canada

**Keywords:** African Americans, carcinoma, diet, micronutrients, renal cell, vitamins

## Abstract

Kidney cancer incidence in African Americans (AA) is higher than among European Americans (EA); reasons for this disparity are not fully known. Dietary micronutrients may have a protective effect on renal cell carcinoma (RCC) development by inhibiting oxidative DNA damage and tumor growth. We evaluated whether any micronutrient associations differed by race in the US Kidney Cancer Study. 1142 EA and AA RCC cases and 1154 frequency‐matched controls were enrolled in a population‐based case‐control study between 2002 and 2007. Dietary micronutrient intake was derived from an interviewer‐administered diet history questionnaire. RCC risk associated with micronutrient intake was estimated using adjusted odds ratios from logistic regression comparing lower to highest quartiles of intake and sample weighting. Inverse associations with RCC risk were observed for α‐carotene, β‐carotene, lutein zeaxanthin, lycopene, vitamin A, folate, thiamin, vitamin C, α‐tocopherol, β‐tocopherol, γ‐tocopherol, and selenium. A trend for β‐cryptoxanthin was suggested among EA but not AA or the total sample (*P‐*interaction = .04). Otherwise, findings did not differ by race, gender, age, or smoking status. The increase in RCC risk associated with lower micronutrient intake is similar within AA and EA populations. A diet rich in sources of micronutrients found in fruits, vegetables, and nuts may help to reduce the overall risk of RCC.

## 1 INTRODUCTION

It is estimated that 63 990 new cases of kidney cancer (KCa) will be identified in 2017, making it the sixth leading cancer diagnosed in men and tenth for women in the United States.^1^ Incidence rates have been rising over past decades, with rates two times higher among men than women, and approximately 20% higher among African Americans (AA) compared to European Americans (EA).^2^ Higher incidence rates, lower survival rates, and earlier age at diagnosis suggest that distinct factors may influence the development of kidney cancer within AA.^3^

Renal cell carcinoma (RCC) makes up approximately 90% of kidney cancer cases, and risk factors include cigarette smoking, obesity, hypertension, and alcohol.^2^, ^4^, ^5^, ^6^, ^7^, ^8^, ^9^, ^10^, ^11^ Racial differences in the distribution of several of these risk factors likely account for some of the excess risk observed in AA as compared to EA populations.^4^, ^12^, ^13^ Many vitamins from diet and supplements, including vitamin A, C, and B, have been shown to have antioxidative effects and may influence RCC risk.^5^, ^6^, ^7^, ^8^, ^14^, ^15^ Intake of dietary E vitamins, including α‐, β‐, δ‐, and γ‐tocopherol, is suggested to be chemo‐preventative.^16^ Supplementation with selenium may influence RCC risk, and selenium has been shown to possess anti‐tumor functions.^17^, ^18^, ^19^, ^20^ Diets rich in carotenoids, including α‐carotene, β‐carotene, β‐cryptoxanthin, lutein zeaxanthin, and lycopene, have been associated with lower RCC risk.^6^, ^21^, ^22^, ^23^ Several studies, however, have demonstrated no significant relationship between micronutrient intake and RCC.^7^, ^14^, ^15^ These discrepancies in the literature highlight the importance of further exploration of the role of micronutrients in RCC risk.

Further, it is unknown whether micronutrient intake explains some of the racial disparity in RCC risk. The objectives of this study were to investigate associations between intake of specific micronutrients and RCC risk, and to evaluate whether any of these associations differed by race in the US Kidney Cancer Study.

## 2 PATIENTS AND METHODS

### 2.1 Study population

The US Kidney Cancer Study is a population‐based case‐control study designed to examine racial disparities between AA and EA in RCC risk. The study was conducted in the metropolitan areas of Detroit (Wayne, Oakland, and Macomb counties), Michigan and Chicago (Cook County), Illinois.^4^ The study was approved by the Institutional Review Boards (IRB) of all participating institutions. Eligible cases were EA and AA men and women between the ages of 20 and 79 years with newly diagnosed RCC. In Chicago, cases were identified via pathology report review at hospitals in Cook County with diagnosis dates between January 1 and December 31, 2003. In Detroit, cases were ascertained through the Metropolitan Detroit Cancer Surveillance System (MDCSS), a participating registry of the National Cancer Institute's Surveillance, Epidemiology, and End Results (SEER) program, with diagnosis dates between February 1, 2002 and July 31, 2006 for EA, and through January 31, 2007 for AA. Controls were identified using motor vehicle records (20‐65 years old) and Medicare eligibility files (65‐79 years old). A detailed description of the sampling strategy is provided Colt et al.^4^ Controls were frequency matched to cases 2:1 in AA and 1:1 in EA on study center, race, sex, and five‐year age categories.

We identified 1918 eligible cases. Of these, 171 died before contact or interview, 92 could not be located, 21 relocated, and we were unable to obtain physician permission to contact 63. We also identified 2718 potentially eligible controls, and were not able to contact 41 due to death before contact or interview, 345 could not be located, and 63 relocated. In total, 1217 cases (77% contacted) and 1235 controls (54% contacted) completed an in‐person interview. We excluded 8 cases and 11 controls who did not complete the Diet History Questionnaire (DHQ) portion of the interview, 16 cases and 18 controls with questionable data quality as reported by the interviewer, and 51 cases and 52 controls with implausible reported total daily caloric intake (TDCI, <800 kcal or >5000 kcal). There were 1142 cases (72.7%) and 1154 controls (50.9%) with data appropriate for inclusion in these analyses.

### 2.2 Data collection

All participants gave informed consent prior to data collection. In‐person computer‐assisted personal interviews were conducted for each participant by trained personnel. Information was collected on demographics and past medical history, including hypertension, height and weight, diet, and smoking.

Dietary micronutrient intake was assessed using a modified version of the NCI DHQ^24^, which asked about usual adult diet prior to two years ago. The DHQ was interviewer‐administered and queried frequency of intake for foods, beverages, multivitamin, vitamin C, and vitamin E supplements, for a total of 80 line items. Portion sizes of beverages were reported by subjects, while standard portion sizes were assumed for foods to reduce respondent burden.^25^ Micronutrient intakes were calculated from DHQ responses based on the 1994‐1996 United States Department of Agriculture's Continuing Survey of Food Intake by Individuals (CSFII) with additional nutrients derived from the Nutrition Data Systems for Research (NDS‐R) database (University of Minnesota, Minneapolis, MN).^26^, ^27^ When both CSFII and NDS‐R calculated intake estimates were available for a given micronutrient, the NDS‐R values were used as these assessments are more comprehensive for micronutrient groups.^28^

### 2.3 Statistical analysis

The distributions of geographic region, sex, education, age, smoking status, body mass index (BMI), RCC family history, hypertension history, alcohol intake, and total daily caloric intake (TDCI) were described by counts and weighted percentages for cases and controls within racial category. Daily micronutrient intake (mg/day) was categorized into quartiles based on control distributions; the three lower quartiles were each compared to the highest quartile.

Associations between individual micronutrients and RCC risk were assessed using unconditional logistic regression models to estimate odds ratios (ORs) and 95% confidence intervals (CIs) from adjusted models. These analyses were weighted using a jackknife replication method (Jackknife 2).^29^, ^30^ Sample weights were developed to reduce bias from several sources, including differential sampling rates, survey nonresponse, and deficiencies in the population at risk arising from sampling frames. Sample weights for controls included a poststratification adjustment, so that weighted distribution of controls was equivalent to weighted distribution of cases. Poststratification adjustment also reduced the variability of weights.^31^

The linear trend was tested by analyzing an ordinal variable for micronutrient quartiles in an adjusted model. Adjusted models included variables for: region (Detroit, Chicago), sex (male, female), race (White, African American), age (20‐44, 45‐54, 55‐64, 65‐74, 75+), education (<12 years, high school graduate, some college, 4+ years of college), smoking history (never, occasional/unknown, former, current), BMI (<25, 25‐30, 30‐<35, 35+_kg/m^2^), RCC family history (none with cancer, unknown if diagnosed with cancer, diagnosed with other type of cancer, diagnosed with kidney cancer), and TDCI (continuous variable). Stratum‐specific analyses were also conducted within race, sex, BMI, hypertension status, and smoking history categories. Interaction between stratification variables and each micronutrient was assessed by including an interaction term in the adjusted models. Analyses were also conducted in the subset of clear cell RCC cases (n = 791) and all controls. The acceptable type I error rate was set at α = 0.05, and tests were two‐sided. The tests for linear trend were corrected for multiple comparisons using the Benjamini and Hochberg (1995) method considering the 18 micronutrients evaluated.^32^

## 3 RESULTS

Sociodemographic and baseline health characteristics and nutrient intake levels of 1142 cases and 1154 controls in the eligible study population are presented by race in Table 1. Compared to controls, cases were more likely to report a lower education level, smoke, have a higher BMI and family history of RCC, and diagnosed with hypertension. There were no differences between cases and controls in terms of region, sex, age, or TDCI. EA cases differed from EA controls on education level, smoking status, BMI, and hypertension. AA cases were more likely than AA controls to be male, have a lower education level, higher BMI, hypertension history, and greater caloric intake (Table 1).

**Table 1:** Weighted results of baseline characteristics by case‐control status by race in the US kidney cancer study cohort

	White		African American	
	Cases	Controls	Cases	Controls
	N = 825	N = 691	N = 317	N = 463
Region: N (%)				
Chicago	113 (15.1)	98 (16.1)	70 (21.3)	87 (21.0)
Detroit	712 (84.9)	593 (83.9)	247 (78.7)	376 (79.0)
Sex: N (%)				
Male	477 (61.9)	425 (61.4)	194 (60.4)	211 (58.8)
Female	348 (38.1)	266 (38.6)	123 (39.6)	252 (41.2)
Education: N (%)				
< 12 years	98 (12.6)	62 (9.3)	81 (26.8)	71 (15.8)
High school graduate	301 (36.2)	203 (30.2)	93 (29.1)	159 (33.8)
Some college	208 (24.9)	182 (26.1)	102 (31.2)	160 (33.3)
4+ Years of college	218 (26.3)	244 (34.4)	41 (12.9)	73 (17.1)
Age (years): N (%)				
20‐44	104 (10.3)	90 (10.2)	37 (12.0)	74 (11.0)
45‐54	179 (20.2)	144 (20.5)	90 (26.3)	116 (27.2)
55‐64	246 (29.0)	198 (28.8)	100 (29.5)	131 (30.6)
65‐74	211 (27.9)	192 (28.4)	74 (24.7)	113 (23.4)
75+	85 (12.6)	67 (12.1)	16 (7.5)	29 (7.8)
Smoking status: N (%)				
Never	299 (35.9)	279 (39.9)	108 (34.0)	169 (35.4)
Occasional/Unknown	31 (4.0)	24 (3.4)	17 (6.0)	26 (5.4)
Former	295 (36.9)	271 (39.8)	96 (30.0)	147 (33.5)
Current	200 (23.2)	117 (17.0)	96 (29.9)	121 (25.7)
Body mass index (kg/m^2^): N (%)				
<25	170 (20.0)	207 (29.0)	58 (18.2)	129 (27.2)
25‐30	294 (36.6)	289 (42.8)	114 (37.2)	182 (41.5)
30‐<35	204 (25.2)	123 (18.2)	80 (24.3)	81 (17.4)
35+	150 (17.4)	70 (9.9)	62 (19.2)	66 (13.1)
Unknown	7 (0.9)	2 (0.2)	3 (1.1)	5 (0.7)
Family history of kidney cancer: N (%)				
None with cancer	323 (38.4)	272 (38.1)	160 (50.6)	252 (52.8)
Unknown if diagnosed with cancer	4 (0.6)	5 (0.8)	6 (1.9)	4 (0.9)
Diagnosed with other type of cancer	466 (57.0)	401 (59.2)	135 (42.5)	198 (44.7)
Diagnosed with kidney cancer	32 (3.9)	13 (1.9)	16 (5.1)	9 (1.5)
Ever diagnosed with high blood pressure: N (%)				
No	389 (44.9)	432 (61.3)	92 (29.3)	247 (52.2)
Yes	423 (53.4)	254 (37.9)	222 (69.8)	213 (47.0)
Unknown	13 (1.7)	5 (0.8)	3 (0.9)	3 (0.8)
Daily alcohol intake				
None	159 (19.3)	104 (15.2)	80 (25.9)	127 (26.1)
>0‐0.14 drinks per day	267 (31.3)	215 (31.1)	84 (27.0)	114 (21.9)
>0.14‐0.86 drinks per day	163 (20.2)	157 (22.1)	59 (18.6)	111 (25.9)
>0.86 drinks per day	236 (29.1)	215 (31.6)	92 (27.9)	111 (26.2)
Unknown	‐	‐	2 (0.6)	‐
Total caloric intake: Mean (SE)	2345 (27)	2306 (27)	2609 (51)	2467 (56)
Daily micronutrient intake: Mean (SE)				
α‐carotene (mcg/day)	583 (13)	637 (15)	444 (21)	502 (23)
β‐carotene equivalents (mcg/day)	3268 (71)	3619 (86)	3827 (143)	4295 (156)
β‐cryptoxanthin (mcg/day)	143 (3.6)	145 (2.9)	219 (7.8)	211 (7.9)
Lutein Zeaxanthin (mcg/day)	2611 (69)	2849 (75)	3304 (125)	3628 (129)
Lycopene (mcg/day)	4523 (80)	4609 (95)	3553 (143)	3602 (116)
Vitamin A (mcg/day)	790 (13)	822 (12)	802 (23)	827 (22)
Folate Equivalents (mcg/day)	536 (6.7)	558 (7.0)	597 (14.0)	585 (12.7)
Niacin (mg/day)	27.3 (0.3)	27.6 (0.3)	28.8 (0.8)	27.5 (0.6)
Riboflavin (mg/day)	2.3 (0.03)	2.3 (0.03)	2.3 (0.05)	2.2 (0.05)
Thiamin (mg/day)	1.7 (0.02)	1.8 (0.02)	2.0 (0.04)	1.9 (0.04)
Vitamin B6 (mg/day)	2.1 (0.03)	2.2 (0.02)	2.4 (0.06)	2.3 (0.05)
Vitamin B12 (mg/day)	5.6 (0.08)	5.5 (0.08)	5.5 (0.14)	5.2 (0.13)
Vitamin C (mg/day)	335 (8)	384 (11)	366 (15)	375 (13)
α‐Tocopherol (mg/day)	8.5 (0.12)	9.0 (0.12)	8.7 (0.25)	8.8 (0.21)
β‐Tocopherol (mg/day)	0.3 (0.005)	0.3 (0.005)	0.4 (0.010)	0.3 (0.008)
δ‐Tocopherol (mg/day)	2.6 (0.05)	2.7 (0.05)	2.8 (0.09)	2.7 (0.07)
γ‐Tocopherol (mg/day)	17.9 (0.26)	18.2 (0.29)	18.7 (0.53)	18.3 (0.46)
Selenium (mcg/day)	116.8 (1.5)	117.0 (1.3)	129.4 (3.1)	123.3 (2.7)

**Figure 1:**
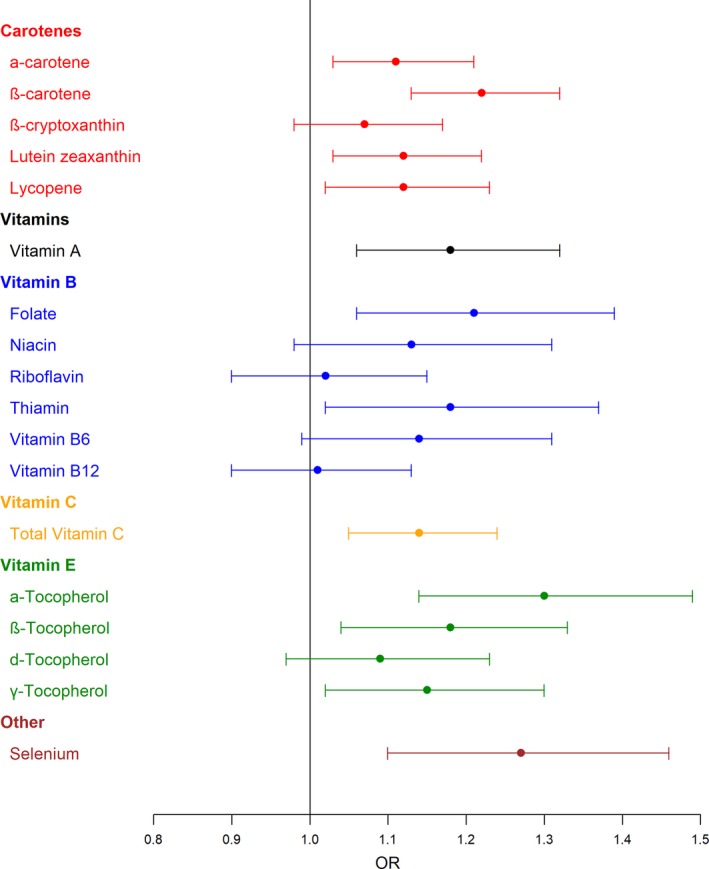
Odds ratios, with 95% confidence intervals, from multivariate logistic regression models of association between micronutrients and renal cell carcinoma in the US Kidney Cancer Study cohort. OR: Adjusted for total calories, region, sex, race, age, education, smoking history, BMI, family history of RCC, hypertension, and alcohol intake; micronutrients are included as an ordinal variable for quartiles of intake in the controls.

Inverse associations with RCC risk were observed for α‐carotene (*P*‐trend = .021), β‐carotene (*P*‐trend <.001), lutein zeaxanthin (*P*‐trend = .021), and lycopene (*P*‐trend = .036) among all participants after adjustment for all variables (Table 2, Figure 1). While β‐cryptoxanthin intake was not significantly associated with RCC risk, we did observe a significant interaction with race (*P‐*interaction = .040), with an inverse association suggested among EA (*P*‐trend = .102), but no association observed among AA (*P*‐trend = .649, Table 2).

**Table 2:** Weighted associations overall and by race between micronutrient intake and renal cell carcinoma (RCC) in the US kidney cancer study cohort

Micronutrient	All Participants	Associations by Race		
	OR^a^ (95% CI)	White	African American	*P*‐interaction
		OR^b^ (95% CI)	OR^b^ (95% CI)	
Carotenes				
α‐carotene				
>754 mcg/day	1	1	1	.16
468‐754 mcg/day	1.0 (0.8‐1.3)	1.0 (0.8‐1.4)	0.8 (0.4‐1.5)	
267‐467 mcg/day	1.4 (1.1‐1.8)	1.3 (1.0‐1.8)	1.6 (0.9‐3.0)	
<=266 mcg/day	1.3 (1.0‐1.6)	1.1 (0.8‐1.5)	1.8 (1.1‐3.1)	
*P*‐trend (FDR corrected)	.021	.232	.024	
β‐carotene equivalents				
>4936 mcg/day	1	1	1	.92
3275‐4936 mcg/day	1.1 (0.8‐1.5)	1.1 (0.8‐1.5)	1.3 (0.8‐2.3)	
2121‐3274 mcg/day	1.5 (1.2‐2.0)	1.5 (1.1‐2.1)	1.6 (0.9‐2.6)	
<=2120 mcg/day	1.8 (1.4‐2.3)	1.6 (1.2‐2.3)	2.3 (1.3‐3.9)	
*P*‐trend (FDR corrected)	<.001	.004	.024	
β‐cryptoxanthin				
>216 mcg/day	1	1	1	.04
146‐216 mcg/day	0.8 (0.6‐1.1)	0.8 (0.6‐1.1)	0.9 (0.5‐1.4)	
93‐145 mcg/day	1.0 (0.8‐1.4)	1.1 (0.7‐1.5)	0.9 (0.6‐1.4)	
<=92 mcg/day	1.1 (0.8‐1.5)	1.2 (0.9‐1.7)	0.9 (0.5‐1.5)	
*P*‐trend (FDR corrected)	.174	.102	.649	
Lutein Zeaxanthin				
>4014 mcg/day	1	1	1	.91
2480‐4014 mcg/day	1.1 (0.8‐1.5)	1.1 (0.8‐1.6)	1.2 (0.8‐1.9)	
1716‐2479 mcg/day	1.1 (0.8‐1.4)	1.0 (0.7‐1.4)	1.4 (0.8‐2.3)	
<=1715 mcg/day	1.5 (1.1‐2.0)	1.4 (1.0‐2.0)	1.7 (1.1‐2.9)	
*P*‐trend (FDR corrected)	.021	.102	.074	
Lycopene				
>5368 mcg/day	1	1	1	.95
3900‐5368 mcg/day	1.1 (0.9‐1.4)	1.2 (0.9‐1.5)	0.8 (0.4‐1.4)	
2527‐3899 mcg/day	1.4 (1.1‐1.8)	1.4 (1.1‐1.9)	1.1 (0.6‐1.8)	
<=2526 mcg/day	1.3 (1.0‐1.8)	1.2 (0.8‐1.7)	1.5 (0.8‐2.8)	
*P*‐trend (FDR corrected)	.036	.163	.109	
Vitamins				
Vitamin A				
>1041 rae ‐ mcg/day	1	1	1	.93
761‐1041 rae ‐ mcg/day	1.6 (1.2‐2.1)	1.6 (1.2‐2.3)	1.5 (0.9‐2.6)	
548‐760 rae ‐ mcg/day	1.4 (1.1‐2.0)	1.4 (1.0‐2.0)	1.8 (1.0‐3.1)	
<=547 rae ‐ mcg/day	1.9 (1.3‐2.6)	1.8 (1.2‐2.7)	2.2 (1.1‐4.4)	
*P*‐trend (FDR corrected)	.015	.077	.068	
B Vitamins				
Dietary folate equivalents				
>689 mcg/day	1	1	1	.79
542‐689 mcg/day	1.1 (0.8‐1.5)	1.1 (0.8‐1.5)	1.2 (0.7‐2.1)	
418‐541 mcg/day	1.4 (1.0‐2.0)	1.4 (0.9‐2.1)	1.5 (0.8‐2.9)	
<=417 mcg/day	1.7 (1.1‐2.6)	1.6 (1.0‐2.6)	2.4 (1.2‐4.9)	
*P*‐trend^c^	.021	.102	.062	
Niacin				
>33 mg/day	1	1	1	.91
28‐33 mg/day	1.2 (0.8‐1.6)	1.1 (0.8‐1.6)	1.3 (0.7‐2.2)	
21‐27 mg/day	1.4 (1.0‐2.0)	1.3 (0.9‐2.0)	1.9 (1.0‐3.7)	
<=20 mg/day	1.4 (0.9‐2.2)	1.3 (0.8‐2.2)	2.3 (1.0‐5.2)	
*P*‐trend^c^	.117	.294	.074	
Riboflavin				
>2.7 mg/day	1	1	1	.53
2.2‐2.7 mg/day	1.0 (0.7‐1.3)	0.9 (0.6‐1.2)	1.2 (0.7‐2.0)	
1.7‐2.1 mg/day	1.0 (0.7‐1.4)	1.0 (0.7‐1.5)	0.9 (0.5‐1.6)	
<=1.6 mg/day	1.0 (0.7‐1.5)	1.0 (0.6‐1.6)	1.2 (0.6‐2.4)	
*P*‐trend^c^	.850	.858	.889	
Thiamin				
>2.2 mg/day	1	1	1	.50
1.9‐2.2 mg/day	1.2 (0.9‐1.6)	1.2 (0.8‐1.6)	1.3 (0.8‐2.1)	
1.5‐1.8 mg/day	1.5 (1.0‐2.2)	1.4 (0.9‐2.2)	2.0 (1.1‐3.6)	
<=1.4 mg/day	1.6 (1.0‐2.6)	1.6 (0.9‐2.8)	1.6 (0.8‐3.5)	
*P*‐trend^c^	.046	.131	.179	
Vitamin B6				
>2.7 mg/day	1	1	1	.59
2.2‐2.7 mg/day	1.1 (0.7‐1.5)	0.9 (0.6‐1.3)	1.8 (1.1‐3.2)	
1.7‐2.1 mg/day	1.4 (0.9‐2.0)	1.2 (0.8‐1.9)	2.0 (1.1‐3.4)	
<=1.6 mg/day	1.4 (0.9‐2.2)	1.3 (0.7‐2.2)	1.8 (0.9‐3.8)	
*P*‐trend^c^	.109	.220	.173	
Vitamin B12				
>6.6 mg/day	1	1	1	.39
5.1‐6.6 mg/day	1.1 (0.8‐1.5)	1.2 (0.8‐1.7)	0.9 (0.6‐1.4)	
3.7‐5.0 mg/day	1.1 (0.8‐1.5)	1.2 (0.8‐1.8)	0.9 (0.5‐1.5)	
<=3.6 mg/day	1.0 (0.7‐1.5)	1.0 (0.7‐1.6)	1.0 (0.5‐1.8)	
*P*‐trend^c^	.889	.858	.889	
Vitamin C				
Total Vitamin C (Dietary + Supplements)				
>666 mg/day	1	1	1	.09
255‐666 mg/day	1.4 (1.1‐1.7)	1.4 (1.1‐1.8)	1.3 (0.8‐2.1)	
145‐254 mg/day	1.4 (1.1‐1.9)	1.4 (1.0‐2.0)	1.5 (1.0‐2.3)	
<=144 mg/day	1.6 (1.2‐2.0)	1.7 (1.3‐2.3)	1.1 (0.7‐1.8)	
*P*‐trend^c^	.011	.014	.575	
Vitamin E				
Dietary α‐Tocopherol				
>11.0 mg/day	1	1	1	.98
8.6‐11.0 mg/day	1.4 (1.1‐1.9)	1.4 (1.0‐1.9)	1.6 (0.9‐2.9)	
6.5‐8.5 mg/day	1.7 (1.2‐2.4)	1.5 (1.0‐2.2)	2.8 (1.4‐5.5)	
<=6.4 mg/day	2.3 (1.6‐3.4)	2.1 (1.4‐3.4)	3.6 (1.6‐8.0)	
*P*‐trend^c^	.001	.024	.021	
Dietary β‐Tocopherol				
>0.42 mg/day	1	1	1	.95
0.33‐0.42 mg/day	1.5 (1.1‐1.9)	1.6 (1.2‐2.2)	1.0 (0.6‐1.7)	
0.25‐0.32 mg/day	1.4 (1.0‐1.8)	1.4 (1.0‐1.9)	1.3 (0.7‐2.7)	
<=0.24 mg/day	1.8 (1.2‐2.7)	1.8 (1.2‐2.9)	2.1 (1.0‐4.4)	
*P*‐trend^c^	.023	.102	.097	
Dietary δ‐Tocopherol				
>3.29 mg/day	1	1	1	.41
2.41‐3.29 mg/day	1.0 (0.8‐1.3)	1.0 (0.8‐1.4)	0.8 (0.5‐1.3)	
1.69‐2.40 mg/day	1.3 (0.9‐1.7)	1.2 (0.8‐1.7)	1.7 (1.0‐2.9)	
<=1.68 mg/day	1.2 (0.8‐1.8)	1.3 (0.8‐2.0)	1.2 (0.6‐2.2)	
*P*‐trend^c^	.186	.253	.285	
Dietary γ‐Tocopherol				
>22.6 mg/day	1	1	1	.59
17.0‐22.6 mg/day	1.4 (1.0‐1.8)	1.3 (1.0‐1.8)	1.4 (0.8‐2.6)	
12.7‐16.9 mg/day	1.4 (1.0‐1.9)	1.4 (0.9‐2.0)	1.4 (0.7‐2.6)	
<=12.6 mg/day	1.7 (1.2‐2.4)	1.6 (1.1‐2.5)	2.0 (0.9‐4.3)	
*P*‐trend^c^	.036	.102	.158	
Other				
Selenium				
>146 mcg/day	1	1	1	.35
115‐146 mcg/day	1.2 (0.9‐1.6)	1.1 (0.8‐1.5)	1.9 (1.1‐3.4)	
89‐114 mcg/day	1.7 (1.2‐2.4)	1.6 (1.0‐2.3)	2.4 (1.2‐4.8)	
<=88 mcg/day	2.0 (1.3‐3.0)	1.9 (1.1‐3.1)	2.4 (1.1‐5.3)	
*P*‐trend^c^	.008	.028	.097	

a Adjusted for total calories, region, sex, race, age, education, smoking history, BMI, family history of RCC, hypertension, and alcohol intake.

b Adjusted for total calories, region, sex, age, education, smoking history, BMI, family history of RCC, hypertension, and alcohol intake.

c FDR corrected.

Vitamin A was inversely associated with RCC risk (*P*‐trend = .015). Compared to individuals in the highest quartile, those in the lowest were 1.9‐fold more likely to have increased risk of RCC. For B vitamins, significant inverse associations with RCC were observed for dietary folate (*P*‐trend = .021) and thiamin (*P*‐trend = .046) among all participants. Total vitamin C was significantly inversely associated with RCC risk (*P*‐trend = .011). No significant interactions were observed between race and A, B, or C vitamins. Among E vitamins, α‐tocopherol (*P*‐trend = .001), β‐tocopherol (*P*‐trend = .023), and γ‐tocopherol (*P*‐trend = .036) demonstrated significant inverse association with RCC risk among all participants (Table 2, Figure 1). No significant interactions with race were observed for any of the E vitamins.

Inverse association with RCC risk was also noted for overall selenium intake Table 2, Figure 1). Compared to individuals in the highest quartile, those that reported in the lowest quartile selenium intake had a 2.0‐fold increased risk of RCC (*P*‐trend = .008).

We also performed weighted analyses between micronutrient intake and RCC stratified by sex, BMI, hypertension, and smoking status, and observed similar results (data not shown). Notably, no significant interactions between any micronutrients examined and race, age or other RCC risk factors were observed (Table 2 and data not shown). Results from analyses restricted to the subset of 791 clear cell RCC cases and all controls did not appreciably differ from those in the entire sample (data not shown).

## 4 DISCUSSION

Our analysis of micronutrient intake with risk of RCC by race in a population‐based case‐control study demonstrated significant inverse associations between intake of micronutrients and RCC risk. We discovered these associations are similar by race for both AA and EA, noting stronger protective trends among AA. Despite known differences in RCC risk attributable to gender, age, smoking status, and alcohol, we found that these characteristics did not modify the protective effects of micronutrients.

Carotenoids, such as lycopene and β‐cryptoxanthin, are exclusively obtained from dietary sources that originate from fruits and vegetables, including tomato, watermelon, papaya, and pink grapefruit.^33^ We observed significant inverse associations with RCC risk independent of race for α‐carotene, β‐carotene, lutein zeaxanthin, and lycopene. We observed an interaction between β‐cryptoxanthin intake and race, wherein β‐cryptoxanthin was associated with decreased risk among EA but not AA. Because none of the quartile‐specific ORs among EA were statistically significant, implications for β‐cryptoxanthin consumption recommendations are not clear. Previous reports of associations between carotenoids and RCC are inconsistent. Aligned with our observations, previous case‐control,^6^, ^34^ individual cohort^21^ and pooled cohort^22^ studies have demonstrated significant associations between intake of carotenoids and RCC risk.^34^, ^35^, ^36^ However, other case‐control^37^ and cohort^5^, ^7^, ^8^, ^14^, ^23^ studies did not detect an association. Reasons for discrepancies in findings among dietary risk factor studies may be due to differences in the source populations, variations in dietary assessment methods, and varying degrees of recall bias between studies.

Among the vitamins, we reported significant inverse associations with RCC risk for vitamin A, total vitamin C, dietary folate and thiamin in all participants. By race, we observed significant associations with dietary folate and niacin and RCC among AA. In contrast, total vitamin C intake was significantly associated with risk of RCC among EA. Previous population‐based studies have demonstrated significant inverse associations with vitamin C intake and RCC.^6^, ^35^

Our study is novel as it is the first to assess the association of micronutrient intake, including tocopherols, with RCC risk among AA. Of vitamin E compounds, there are four tocopherols (α‐ β‐, δ‐, and γ‐tocopherol) found naturally in nuts, fish, leafy greens, and vegetable oils. We noted inverse associations between α‐tocopherol and RCC risk among all participants, with greater protective effects among AA. Aligned with these observations, previous studies demonstrated an association between increased intake of tocopherols with decreasing RCC risk for males and females, nonsmokers, and ever smokers.^5^, ^36^, ^37^, ^38^, ^39^, ^40^ In contrast, other studies reported associations of marginal or no significance between RCC risk and tocopherol intake.^7^, ^8^, ^10^, ^14^, ^15^, ^41^

Studies examining the relationships between mineral intake and RCC risk have previously found no associations with selenium.^5^, ^36^ Selenium plays critical roles in major metabolic pathways, and is an essential element to protect the body from harmful substances. Recent studies demonstrated that selenium supplementation reduces side effects from chemotherapy and improves kidney and liver functions in cancer patients.^16^, ^19^, ^20^ Analysis of selenium intake by participants in our study revealed inverse associations with RCC risk for both EA and AA, with greater protective effects among AA. Based on these observations and the findings of Stafford, et al^3^ which identified racial disparities in RCC incidence and survival, additional work is needed to provide insight on the association between micronutrients and RCC development specifically among AA.

Similar to other studies, we found no interactions with obesity or hypertension and micronutrients with respect to RCC risk.^35^ We acknowledge that limitations of this study include self‐reported dietary information and the inherent limitations of a case‐control study design, in particular, low participation rates among controls, typical of population‐based case‐control studies. The use of sample weights helps reduce bias arising from nonresponse, as weights account for differential nonresponse across subgroups (age, sex, and county of residence) for which data were available for both respondents and nonrespondents. We were unable to stratify patients by smoking status due to small sample sizes, but adjusted this in our weighted analyses.

The current study is strengthened by the inclusion of large numbers of AA cases and controls and the use of in‐person interviews. As a result, we were able to examine associations between the micronutrients of interest and RCC risk overall and by race and sex after adjusting for known RCC risk factors. Use of SEER data is another strength as it allowed for a considerable number of pathologically verified cases, and results can be generalized to the larger United States population.

While a strength of the study is the inclusion of adequate numbers of AAs and EAs to examine associations stratified by race, other racial/ethnic groups were not represented thus limiting generalizability to these two race groups. Knowledge of disease status may have biased FFQ responses, resulting in differential misclassification of exposures. Additionally, DHQs have known measurement error which may result in nondifferential misclassification, biasing effect estimates toward the null. The DHQ was modified for length from the validated NCI DHQ;^24^ however, the modified version was not independently validated. The micronutrient values available for analysis only included supplement use for vitamin C variable; the remaining variables did not include micronutrients from supplement sources. We expect that this would result in a bias toward the null in the observed odds ratios. To address this concern, we repeated the analyses with adjustment for multivitamin use, and there was no change in which *P*‐trends were statistically significant or not. Although we adjusted for multiple comparisons, we cannot rule out the role of chance and possibility of false positives, as in any epidemiologic study.

In conclusion, we observed high intake of micronutrients to be associated with lower RCC risk among AA and EA. These findings support a diet rich in carotenes, tocopherols, vitamins, and minerals to help reduce the risk of RCC among both AA and EA. Despite differences in underlying disease risk attributable to race, gender, age, smoking status, and alcohol, these characteristics did not modify the protective associations of micronutrients observed in our study. Although our findings suggest that dietary interventions involving increased consumption of these micronutrients could reduce RCC risk, further investigation is needed before causal inferences can be drawn.

## CONFLICT OF INTERESTS

There are no conflict of interests for any authors.
